# Neural Mechanisms of Social Homeostasis: Dynamic Range Plasticity

**DOI:** 10.1523/JNEUROSCI.0224-25.2025

**Published:** 2026-02-25

**Authors:** Jianna Cressy, Caroline Jia, Jonathan Salk, Kay M. Tye

**Affiliations:** ^1^University of California San Diego, La Jolla, California 92093; ^2^Medical Scientist Training Program (MSTP), University of California San Diego, San Diego, California 92093; ^3^Salk Institute for Biological Studies, La Jolla, California 92037; ^4^Howard Hughes Medical Institute, Salk Institute for Biological Studies, La Jolla, California 92037; ^5^David Geffen School of Medicine, University of California, Los Angeles, California 90095; ^6^Kavli Institute for the Brain and Mind, La Jolla, California 92093

**Keywords:** buffering, exclusion, homeostasis, neuroscience, pain, social

## Abstract

To address the open question of how animals respond to dynamic changes in their social environment, we propose a framework in which cumulative effects of previous experience inform a key mechanism to regulate the bounds within which social experiences can vary without activating costly, corrective mechanisms. We define this as the dynamic range. By leveraging insights from human studies and animal models, future research will contribute to our understanding of the intricate neural underpinnings that mediate social behavior. Key questions facing the field of social neuroscience include the following: How do experienced statistics of the environment calibrate neural circuits that regulate social behavior? What neural mechanisms allow some individuals to better adapt to dynamic environments than others? How does social homeostasis interact with other homeostatic systems, such as the stress response? This framework provides a testable model for investigating the neural mechanisms through which history calibrates the dynamic range within detector, control center, and effector systems of social homeostasis.

## Introduction

Social behavior is dynamically regulated. An individual who seeks out social interaction today may withdraw from the same situation tomorrow. Colloquially, the metaphor of having a “social battery” captures this experience—that it is energetically costly to regulate our social interactions. Traditionally, previous experience has been considered important because it determines the expression of future behavior (Skinner, 1938). The neural systems that dictate the expression of social behavior are shaped by an individual's unique history (Table 1), genetic variation, and the interaction of these factors (Robinson et al., 2008). These interactions generate substantial variability across time, highlighting that social motivation is dynamically regulated (MacDonald et al., 2006; Hariri, 2009; Waschke et al., 2021). The ability to change the expression of one's behavioral repertoire according to one's history and current environment allows animals to optimize their social relationships throughout their lifetimes (Teles et al., 2016). The conditions that lead to this dynamic regulation of social behavior can be explained using the framework of social homeostasis (Table 1).

**Table 1.** Glossary of terms

Term	Definition
Glossary of terms in the context of social homeostasis	
Social homeostasis	Conceptual framework for how we regulate the quantity and quality of social contact
Detector	A neural system that senses changes in the quality and/or quantity of an individual’s current social environment
Control center	A neural system that compares deviations in social utility to the encoded homeostatic set-point to calculate deficits or surpluses of social interaction
Effector	A neural system that drives motivated behavior to resolve deficits and/or surpluses in social utility (e.g., social approach or social avoidance)
Social utility	The product of the detected quality and quantity of an individual’s current social environment
Homeostatic set-point	An individual’s optimal level of social utility at a given point in time
Homeostatic dynamic range	The range between an individual’s upper and lower bounds for triggering effector system activation around the homeostatic set-point
Set-point deviation	Social utility that falls outside of the bounds of the dynamic range (e.g., social deficit or social surplus) and activates the effector system
Set-point shift	The change of an individual’s homeostatic set-point due to failure of the effector system to compensate for set-point deviations
Dynamic range plasticity	The change in the bounds of an individual’s dynamic range in response to experience (e.g., stress, variability in social environment)
History	Previous experiences from an individual’s environment
Valence	Positive or negative emotional quality assigned to a social experience
Enrichment	Social conditions that offer variety in partners, settings, and types of social engagement. (e.g., group housing, varied partners, play)
Social buffering	Social interactions that reduce an individual’s neurophysiological stress response
Tolerance	An individual’s capacity to endure changes in the social environment (e.g., social isolation, overcrowding) without activation of the effector system. Tolerance is directly related to dynamic range width
Resilience	An individual’s ability to maintain coordinated function across detector, control center, and effector systems during and after set-point deviations
Stress load	The aggregate burden of past set-point deviations
Low stress	Stress that remains within the dynamic range, does not activate the effector system, and *narrow*s the dynamic range
Moderate stress	Stress that briefly exceeds the dynamic range, transiently activates the effector system, and *widens* the dynamic range
High stress	Stress that exceeds the dynamic range, exhausts effector activation and set-point shifts, and *narrows* the dynamic range
General glossary of terms	
Heart rate variability	A measure of the variation of time between heartbeats
Stress inoculation	Brief, intermittent and controlled exposure to stressors that builds resilience to future stressors

Homeostasis was a term coined by Walter Cannon to describe the physiological processes by which biological systems maintain stable internal conditions through mechanisms to adapt to changes in the external environment (Cannon, 1939). In the human body, homeostatic systems regulate body temperature, blood glucose, fluid balance, and other physiological parameters through adaptive mechanisms that depend on robust feedback systems (Kiesewetter and Schmiemann, 2022; Libretti and Puckett, 2025).

**Figure 1.**
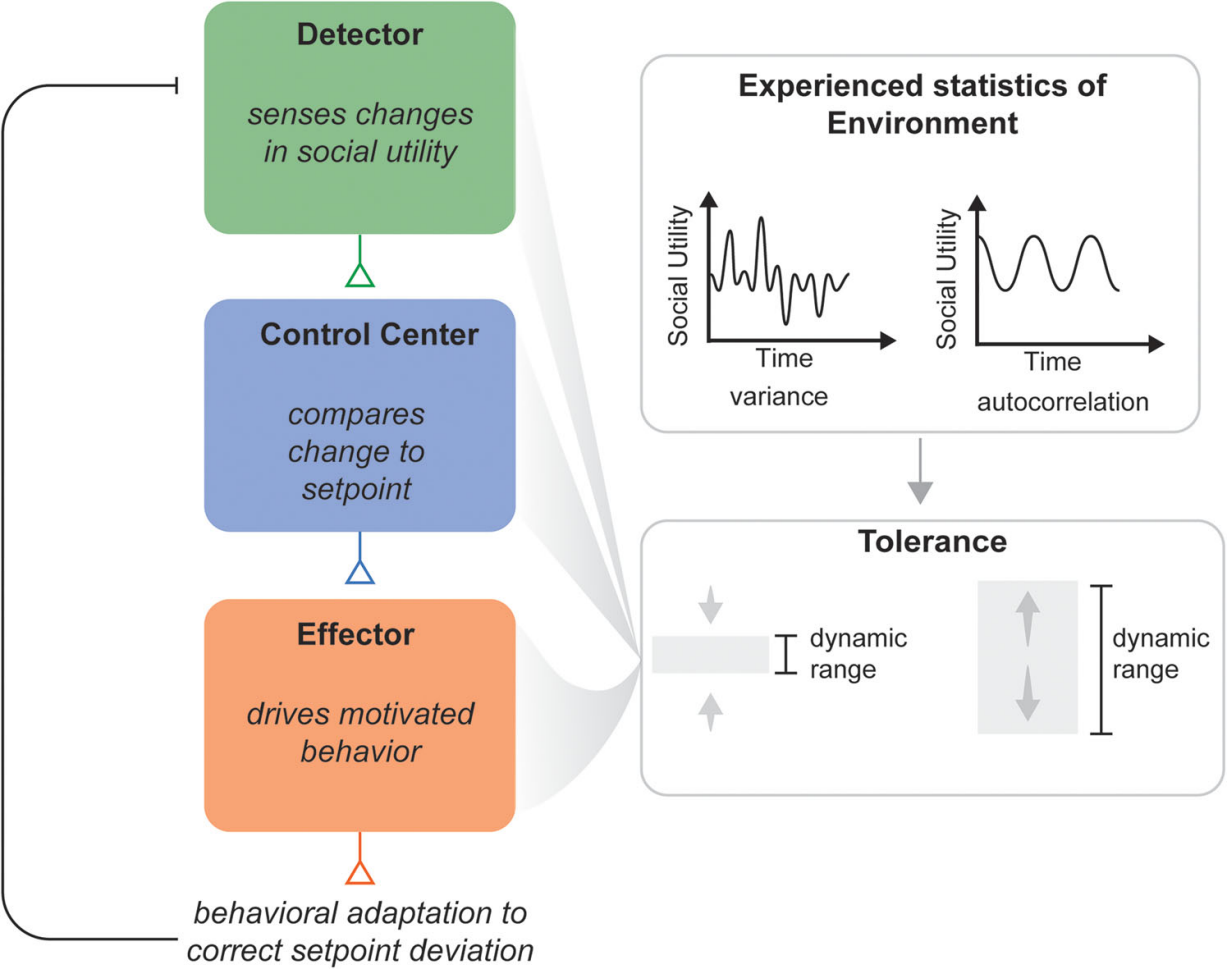
Schematic of a three-component system of social homeostasis shaped by tolerance. Social homeostasis operates through three core components: a detector, which senses current social input and encodes deviations from expected conditions; a control center, which compares input to a social set-point to calculate set-point deviations; and an effector, which generates behavioral or physiological responses to restore balance. Statistical experiences of an individual’s environment shape tolerance, which is parameterized by the dynamic range, which in turn calibrates the detector, control center, or effector components. Adapted from Matthews and Tye, 2019.

**Figure 2.**
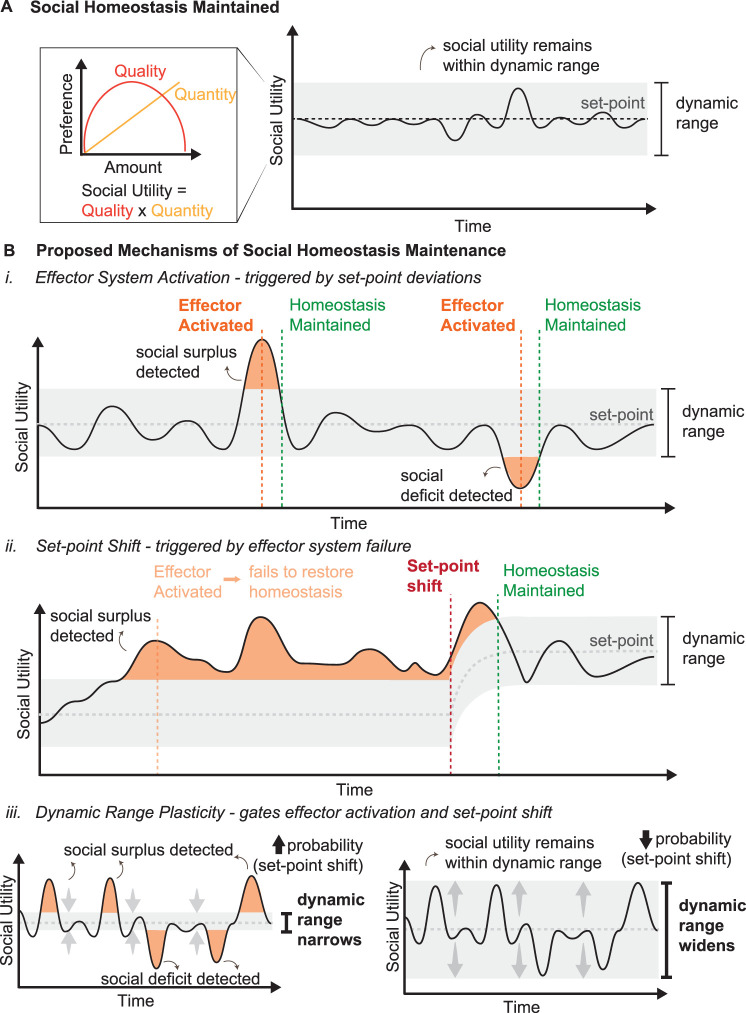
Mechanisms of social homeostasis maintenance. ***A***, Social homeostasis maintained. Social utility is the product of the quality and quantity of social interactions. When social utility remains within the dynamic range of the set-point, social homeostasis is maintained. ***B***, Proposed mechanisms for maintaining social homeostasis. ***i***, Effector system activation. Set-point deviations (social surpluses or deficits) trigger effector system activation that restores social utility to the dynamic range. ***ii***, Set-point shift. Chronic set-point deviations may lead to failure of the effector system to compensate, triggering a shift in set-point. ***iii***, Dynamic range plasticity. The boundaries of the dynamic range can narrow (left) or widen (right), altering tolerance for fluctuations in social utility; when the range widens, larger deviations are absorbed without triggering effector activation or set-point shifts, where a narrow range makes effector system activation and set-point shifts more likely.

The *Social Homeostasis* hypothesis builds upon the principles of physiological homeostasis and posits that organisms continuously monitor their social environment through a *detector system* that senses the quantity and quality of social interactions, integrating this information into a measure of *social utility* (Table 1; Fig. 2*A*). Then, a *control center* compares the detected social contact against an internal reference point, or *set-point*, and adjusts behavior via an effector system to minimize deviations from an optimal range (Table 1, Fig. 1; Matthews and Tye, 2019; Lee et al., 2021). When social contact falls outside of this optimal range of the set-point, called the *dynamic range*, this is considered a *set-point deviation* (social surplus or social deficit), and an effector system is activated to return to the optimal range (Table 1; Fig. 2*Bi*). However, when the effector system fails to bring the system back within the dynamic range, the social set-point shifts to maintain stability (Table 1). This mechanism of homeostatic maintenance is called a *set-point shift* (Table 1; Fig. 2*Bii*). While previous social homeostasis frameworks have focused primarily on these set-point mechanisms, they have largely overlooked the cumulative effects of history on the system. Previously, we have discussed the neural mechanisms that regulate set-point shifts after chronic deficits in social contact, such as social isolation (Lee et al., 2021). Here, we expand this framework to propose a complementary mechanism for homeostatic maintenance: *dynamic range plasticity* (Fig. 2*Biii*).

The *dynamic range* represents the upper and lower bounds around the *set-point* or optimal level of social contact*.* The *dynamic range* is not uniform across an individual’s lifetime, but rather shaped by experience. We propose that *dynamic range plasticity*—the change in the bounds of optimal social contact due to previous experience—is a fundamental mechanism through which individuals maintain *social homeostasis. Dynamic range plasticity* serves two key functions: (1) it adjusts the bounds of tolerable social contact by incorporating the volatility of an individual’s social environment and (2) it gates costly set-point shifts by providing a “buffer zone” that prevents unnecessary adjustments of the system.

The capacity of animals to adapt to changing environments reflects what ecologists define as *tolerance*—the ability of an individual to minimize fitness costs under persistent stress, rather than eliminating the stressor itself (Table 1; Grime and Pierce, 2012). In the framework of social homeostasis, the width of the *dynamic range* determines an individual’s *tolerance*, defined as an individual’s capacity to endure changes in the social environment (e.g., social isolation, overcrowding) without activating the *effector system*. Thus, *tolerance* may be parameterized as the width of the *dynamic range*. In other words, individuals with a wide dynamic range possess greater tolerance, rarely needing to activate the effector system, while those with narrow dynamic range have reduced tolerance and will activate the effector system in response to minor environmental changes (Table 1; Fig. 2*Biii*). When the effector system is activated, an individual’s *resilience*—the system’s ability to maintain functioning and recover after homeostatic responses are triggered—determines how well they navigate and recover from these social disruptions (Table 1).

While set-point shifts can be initially adaptive in a dynamic environment, *repeated* set-point shifts are maladaptive and create physiological “wear-and-tear” on the organism (McEwen, 1998). *Dynamic range plasticity* provides a first line of defense against this high-cost strategy. When this is insufficient to gate effector activation, set-point shifts offer a secondary mechanism for homeostatic maintenance (Table 1). Which of these mechanisms is engaged will likely depend on the intensity, duration, and frequency of environmental challenges (McEwen and Stellar, 1993; McEwen, 2007).

Together, *set-point shifts* and *dynamic range plasticity* represent two complementary mechanisms of homeostatic maintenance that allow social species to regulate their behavior in response to changing environmental demands. This review synthesizes evidence from human and rodent studies demonstrating how previous experience shapes the dynamic range through specific neural mechanisms. We organize candidate neural substrates according to their roles in the detector, control center, and effector nodes of social homeostasis, providing a framework for understanding how experienced statistics (e.g., quantity and quality of social contact) of the social environment shape social behavior. Current models of social homeostasis (Lee et al., 2021; Matthews and Tye, 2019) emphasize set-point shifts and effector system activation. However, a mechanism to prevent the high cost of repeated set-point shifts in dynamic environments has yet to be defined. In this review, we add an additional layer to the social homeostasis model that incorporates *social tolerance* as a parameter represented by the dynamic range of social experiences (Fig. 1).

## Homeostatic Precedents of Dynamic Range Plasticity

Importantly, set-point shifts and dynamic range plasticity are not mutually exclusive mechanisms but are fundamentally connected. Beyond simple feedback regulation, organisms have the capacity to adjust their sensitivity to environmental changes, tuning how much variation they can tolerate before activating costly corrective responses (Sterling and Eyer, 1988). Having a narrow dynamic range increases the likelihood of a set-point shift in response to minor environmental changes. Conversely, a wider dynamic range allows for greater tolerance of changing environmental stimuli and thus makes set-point shifts less likely (Chu et al., 2025).

Classical studies of thermoregulation in soldiers demonstrate this interconnection: undergoing repeated heat exposure not only reduces heart rate and core temperature (set-point shift) but also promotes thermal tolerance to future heat stress evidenced by delayed onset of sweating and improved cardiovascular stability (dynamic range plasticity; Wyndham, 1951; Rowell et al., 1969; Nielsen et al., 1993; Shapiro et al., 1998; Patterson et al., 2004). Similarly, brief, intermittent exposure to cold water immersion (e.g., 3 min head-out immersions at 10–12°C) over the course of several days blunts cold-induced tachycardia and hyperventilation, both of which are features of the involuntary cold-shock response (Tipton et al., 2000; 1998; Eglin et al., 2015; Seeley et al., 2025).

The clinical importance of studying dynamic range in physiological systems is evident in cardiovascular functioning. Heart rate variability, the variation in the time between two heartbeats, is a measure of the cardiovascular system’s dynamic range and serves as a robust predictor of health outcomes (Table 1; Malik and Camm, 1990). High heart rate variability indicates a flexible, resilient system, while reduced variability correlates with increased cardiac risk and mortality (Villareal et al., 2002; Jarczok et al., 2022). This metric improves with exercise, with trained athletes having higher heart rate variability compared with sedentary individuals (Nagai and Moritani, 2004; Ashok et al., 2022). Interestingly, heart rate variability correlates with increased functional connectivity of the amygdala-medial prefrontal cortex circuit and ventromedial prefrontal cortex activity during decision-making tasks (Sakaki et al., 2016; Maier and Hare, 2017). Interventions that increase heart rate variability, such as aerobic exercise and slow (∼0.1 Hz) paced breathing coupled with real-time biofeedback, have been shown to (1) reduce acute stress reactivity (i.e., reduced heart rate/blood pressure, lower self-reported anxiety, reduced salivary cortisol) and (2) speed recovery from experimental stressors including mental arithmetic and cold pressor tests, with accelerated return of heart rate and blood pressure to pre-stress baselines (Stancák et al., 1996; Wells et al., 2012; Goessl et al., 2017; Lachowska et al., 2020; Makaracı et al., 2023; Amekran and El Hangouche, 2024). Together, these data indicate that cardiac dynamic range plasticity reduces autonomic corrective demand, preventing costly corrections to the system in the face of persistent environmental stress.

Building upon these physiological precedents of thermoregulation and cardiovascular control, where dynamic range plasticity minimizes the corrective demand imposed upon these systems, we formalize the role of dynamic range plasticity in the framework of social homeostasis.

## Defining Dynamic Range Plasticity in Social Homeostasis

Social homeostasis operates through monitoring and regulating social contact to maintain optimal functioning (Fig. 1). To achieve this, we propose that there are three mechanisms of maintenance for social homeostasis: effector system activation, set-point shifts, and dynamic range plasticity. Effector system activation occurs when social contact falls outside of the optimal range of social contact, triggering behavioral responses to restore optimal levels of social contact (Fig. 2*Bi*). Set-point shifts allow this system to adapt when social contact chronically deviates from an optimal range (Fig. 2*Bii*). However, this mechanism alone cannot fully account for the flexible, history-dependent nature of social behavior regulation. We propose that dynamic range plasticity represents a complementary mechanism to set-point shifts for reducing the energetic cost of homeostatic maintenance. Dynamic range represents the upper and lower thresholds around the set-point within which social utility can vary without activating the effector system or triggering set-point shifts. Dynamic range *plasticity* refers to the widening or narrowing of the dynamic range in response to social statistics (Fig. 2*Aiii*).

In the framework of social homeostasis, we propose that dynamic range plasticity is (1) a gating mechanism to prevent costly set-point shifts and (2) shaped by experienced statistics of the environment. For dynamic range plasticity to function as an adaptive, history-dependent mechanism within social homeostasis, we assert that the following criteria be met:

1. History-dependent plasticity: The dynamic range adapts to experienced statistics of the environment (i.e., variance and predictability of previous experiences), recalibrating the thresholds that trigger corrective social behaviors.
2. Component-level implementation: Dynamic range is represented within core homeostatic components—either in the detector (altering deviation detection thresholds), the control center (modifying the mechanisms that compute the difference between current levels of social contact and the optimal set-point), or the effector system (adjusting when and how motivated social behaviors are initiated).
3. Behavioral Effects: Change in dynamic range width will directly influence when and how social behaviors are triggered in response to set-point deviations.

## Neural Substrates of Dynamic Range Plasticity

How is the dynamic range encoded by the brain? Social homeostasis has been conceptualized as a three-node system composed of a detector, control center, and effector that together regulate stability in social behavior (Fig. 1; Matthews and Tye, 2019; Lee et al., 2021). We propose that dynamic range plasticity is an emergent property that is represented in mechanistic changes across these nodes. Here, previous experience recalibrates the sensitivity of detectors, alters computations in control centers, and informs how and when the effector system is activated. In this section, we discuss candidate neural mechanisms within each node that implement dynamic range plasticity.

### Detector

The first step within the system of social homeostasis is detecting features of the social environment. Detectors register deviations from the social set-point by monitoring internal and external environmental cues. Thus, candidate mechanisms for dynamic range plasticity within this node would learn the statistical regularities of the environment to tune the *sensitivity* of the detector to these external cues. Social touch provides a concrete example of detector-level calibration. Social touch from familiar partners activates C-fiber mechanoreceptors that increase oxytocin release and projects to posterior insula and related interoceptive networks (McGlone et al., 2014; Peris et al., 2017; Walker et al., 2017; Dagnino-Subiabre, 2022; Elias and Abdus-Saboor, 2022). These C-fiber tactile afferents respond to pleasant social touch but demonstrate *affective habituation*—where slow (1–10 cm/s), repeated, and prolonged exposure to pleasant touch (low-force forearm vibration) decreases reports of subjective pleasantness (Triscoli et al., 2017; Ree et al., 2019; Bendas et al., 2021). More broadly, repeated exposure to predictable challenge, such as repeated restraint stress and exposure to a novel environment, produces habituation of the stress response [i.e., reduced blood adrenocorticotrophic hormone (ACTH), corticosterone], indicating that detector thresholds for aversive cues can be recalibrated by statistical regularities of the environment (Grissom and Bhatnagar, 2009; Matovic et al., 2020). Social contact during fear conditioning has been shown to reduce Fos expression in the lateral amygdala, suggesting that environmental cues can buffer the neural encoding of unpredictable threat (Fuzzo et al., 2015). Collectively, these data provide candidate mechanisms by which experienced statistics of the environment may tune detector system sensitivity.

### Control center

The control center integrates detector input, compares it to the social set-point to compute the delta between the two, and sends information about any set-point deviation to the effector system (Brown and Brüne, 2012; Joiner et al., 2017). Candidate mechanisms for dynamic range plasticity within this node could (1) filter detector input, (2) store set-point deviation history to scale current estimations of predicted correction effort, or (3) gate outputs to the effector system. The medial prefrontal cortex (mPFC) and anterior cingulate cortex (ACC) are key candidates given their role in prediction, valuation, and top-down regulation of limbic circuits (Etkin et al., 2011; Apps et al., 2016). Brief, unpredictable social isolation stress in nonhuman primates has been shown to prevent isolation-induced anhedonia measured using the sucrose preference test and reduce blood cortisol levels by enhanced ACC glucocorticoid receptor expression (Lee et al., 2014). Magnetoencephalography (MEG) shows that cognitive behavioral therapy (CBT) enhances prefrontal recruitment during affective processing of pictures showing threat-relevant events (e.g., mutilations, assaults, weapons), consistent with strengthened top-down control (Adenauer et al., 2011; Lueken et al., 2014). Causally, reactivating an mPFC social memory trace buffers the behavioral effects of contextual fear conditioning (i.e., freezing) even in the absence of a social partner, demonstrating that previous social information encoded in mPFC ensembles can gate high-cost stress responses to future unpredictable stress (Gutzeit et al., 2020). Developmentally, early social experience (i.e., social play) is required for maturation of parvalbumin-interneuron inhibition in mPFC, establishing excitatory inhibitory (E/I) balance that stabilizes predictive control (Bijlsma et al., 2022). Daily rotations of various forms of environmental enrichment (Table 1) (i.e., running wheel, toys, dome, PVC pipe fittings) increases levels of synaptophysin and PSD-95 within the hippocampus and cortex, supporting a synaptic mechanism for encoding statistical regularities of the environment (Frick et al., 2003; Frick and Fernandez, 2003; Nithianantharajah et al., 2004). In terms of neuromodulation, oxytocin (OT) signaling is a key modulator of social valuation and shows robust plasticity with stress and pair bonding (Froemke and Young, 2021). Interestingly, pair bonding decreases the density of oxytocin neurons in the paraventricular nucleus (PVN) of the hypothalamus, and partner separation rescues this density back to prebonding baselines, indicating durable, experience-linked regulation of neuronal density (Sun et al., 2014; Fricker et al., 2023). Together, these data indicate candidate mechanisms by which the control center may filter detector input, scale responses based on previous experience, or gate outputs to the effector system to shape the dynamic range.

### Effector system

Effector systems trigger corrective responses to restore balance when a set-point deviation is detected, spanning behavioral (approach/avoidance, vocalizations) and physiological domains. In this view, effector systems utilize neural mechanisms to translate control center decisions into behavioral and physiological actions. A history of forced swim stress can act as an experiential switch for corticotropin-releasing factor (CRF) signaling in the nucleus accumbens (NAc), converting CRF’s modulation of dopamine from appetitive to aversive and biasing the approach–avoidance policy toward avoidance, supporting that previous experience shifts effector system responses and choice mapping (Lemos et al., 2012). Mesolimbic dopamine is similarly plastic where forced swim stress in female mice remodels ventral tegmental area (VTA) projections to NAc, enhances optogenetically evoked dopamine (DA) release, and transforms photoactivation of these VTA-DA neurons from a social to an antisocial signal (Wichmann et al., 2017). In terms of neuromodulation, opioids amplify the hedonic value of social interaction via μ-receptors, endocannabinoids promote social interaction through acting on the basolateral amygdala (BLA), and norepinephrine sculpts play via bottom-up networks by acting on α2-adrenergic receptors, providing convergent levers that could raise or lower effector engagement to the same social inputs (Panksepp, 1998; Trezza et al., 2011; 2012; Manduca et al., 2016; Siviy, 2016; Vanderschuren et al., 2016; Zhao et al., 2020). At the synaptic level, social contact after footshock stress dampens neural responsiveness via synaptic metaplasticity in corticotropin-releasing hormone (CRH) neurons, where partner presence buffers stress-induced short-term potentiation (STP) in response to high-frequency afferent stimulation at glutamate synapses (Sterley et al., 2018). Within the hypothalamic-pituitary-adrenal (HPA) axis, a critical mediator of stress adaptation (Keeney et al., 2006; McEwen, 2007; Joëls and Baram, 2009), repeat stress decreases CRH neuron excitability via structural plasticity of the neural membrane surface area (Matovic et al., 2020) and engages oxytocin-CRH cross talk in the PVN (Pati et al., 2022; 2020). At the systems level, higher vagal tone is linked to stronger amygdala–mPFC coupling, consistent with more selective engagement of action-selection circuitry rather than indiscriminate responding (Park and Thayer, 2014; Sakaki et al., 2016; Forte et al., 2019; Magnon et al., 2022). The nuclei of the basal ganglia function as a centralized selection mechanism that resolves competition among motor programs by combining broad tonic inhibition with focal disinhibition (Mink, 1996). Stimulation of the subthalamic nucleus (STN) reverses mPFC control over decision thresholds, consistent with effector feedback within the homeostatic circuit (Cavanagh et al., 2011). Overall, this framework highlights that dynamic range plasticity likely is not localized to a single brain region but emerges from a distributed neural network. By embedding the cumulative influence of previous experience into each node of social homeostasis, we hypothesize that these neural mechanisms may provide clues for how the brain can flexibly shape the dynamic range.

## Experienced Statistics of the Social Environment Shapes the Dynamic Range

The capacity for adaptive social behavior is not fixed but recalibrated by the statistical structure of previous encounters (Sachser et al., 2013; George et al., 2023). We assert that the social brain operates as an experience-dependent estimator (Devaine et al., 2014), continuously adjusting the thresholds that define social tolerance based on two key parameters of previous experience: (1) variability of environmental input (defined as variance and predictability of past events) and the (2) cumulative stress load (Table 1; Selye, 1975; McEwen, 2007; Koolhaas et al., 2011).

From humans to rodents, animals reared in unpredictable environments develop robust capacities for social cohesion, behavioral flexibility, and resilience (Ozbay et al., 2007; Schmid et al., 2014; Dooley et al., 2017; Hartmann and Schmidt, 2020; Linkov et al., 2022; Ameca et al., 2023; Winiarski et al., 2025). Human studies demonstrate that sampling of volatile social contexts, including unstable immigration status and parental loss, enhances cognitive flexibility (Martindale, 1972; Goertzel et al., 1978; Ritter et al., 2012). In a one-armed bandit task where individuals choose between two differently colored rectangles for a monetary reward (£10 or £20), participants show increased learning rate during volatile phases of the experiment to flexibly adapt to changing task rules (when reward probabilities for the two stimuli switch; Behrens et al., 2007). Further, individuals' estimates of task volatility are reflected in the fMRI signal in the ACC when each trial outcome is observed. Accordingly, laboratory measures of flexibility index the capacity to update representations when contingencies shift. In humans, cognitive flexibility can also be quantified using tasks such as the *Unusual Uses Task* (Guilford, 1967), in which participants are asked to generate as many uses as possible for common objects (e.g., a brick) within a short time window. Cognitive flexibility is scored by counting the number of distinct categorical domains represented in their responses (e.g., “weapon,” “tool,” “toy”). Interestingly, individuals that engage with expectation-violating scenarios (e.g., virtual reality environments where objects defy gravity) demonstrate greater cognitive flexibility compared with passive observers, suggesting that active engagement is critical to integrating statistical patterns of past experience into behavioral policies (Ritter et al., 2012). Interestingly, forms of social play that are unpredictable in sequence and outcome, such as rough-and-tumble play in rats (interleaved, bouts of variable duration of pinning, chasing, wrestling, role-switching behaviors), and self-directed pretend play in children, require rapid adjustment to shifting roles and are a natural training ground for self-regulation and cognitive flexibility (Coplan et al., 1994; Pellegrini and Smith, 1998; Gray, 2011; Barker and Munakata, 2015). These effects engage orbitofrontal cortex (OFC) and prefrontal cortex (PFC), which are critical nodes for cognitive flexibility (Nelson and Guyer, 2011), through stimulus valuation (Padoa-Schioppa, 2007; Tom et al., 2007; Cunningham et al., 2011) and integration of complex sequences of stimulus and temporal dynamics (Nogueira et al., 2017; Lin and Zhou, 2024).

Conversely, too little stress may narrow the dynamic range and make an individual more fragile in the face of stress challenges. For example, controlled and highly predictable environments increase rates of anxiety and depression (Niemann-Mirmehdi et al., 2019). Overprotective parenting prevents the development of autonomous coping strategies to mildly stressful tasks such as challenging puzzles and public speaking, has been consistently linked to increased anxiety and social withdrawal (Parker et al., 1979; Muris et al., 2003; Fox et al., 2005; McLeod et al., 2007; Rubin et al., 2009; Jinyao et al., 2012; Clarke et al., 2013), and would likely narrow the dynamic range in our model. Neuroimaging reveals that maternal overprotection correlates with reduced hippocampal volume (Wang et al., 2017), increased amygdala reactivity to social threat, and decreased white matter tract integrity (Farber et al., 2019). Similarly, nonhuman primates exhibit reduced PFC development, diminished prosocial behavior, and poor stress coping (less grooming, fewer number of social approaches) following alterations in social group size (Sallet et al., 2011). Together, these findings support the notion that a history of variability in social experiences builds flexible, well-regulated social behavior, whereas unvarying predictability leads to functional deficits.

## Cumulative Stress Load Shapes the Dynamic Range

**Figure 3.**
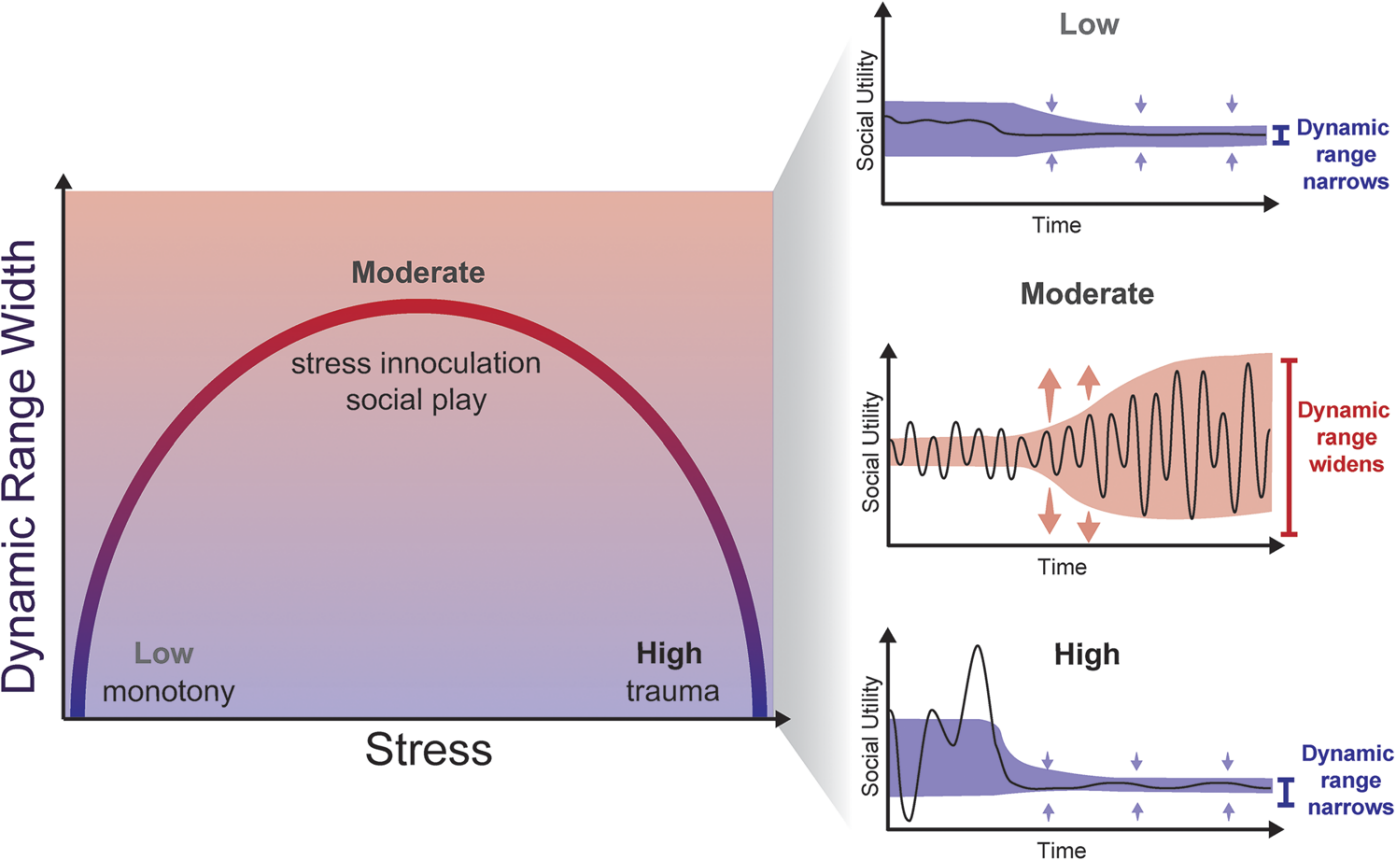
An inverted relationship between stress load and dynamic range. An inverted U relationship between stress and dynamic range width illustrates the concept that environmental experience can optimize the dynamic range. Low stress (monotony, isolation) and high stress (trauma) both induce dynamic range narrowing. In contrast, moderate stressors (stress inoculation, social play) fall within a moderate stress zone that induces dynamic range widening.

Beyond variability, social tolerance is also shaped by cumulative stress load—the aggregate burden of past set-point deviations. Building on Selye's classical theory of eustress (Selye, 1975), we propose that the relationship between stress load and dynamic range can be modeled with an inverted U-shaped curve, where moderate stress (Table 1) expands the dynamic range while low and high stress narrow it (Fig. 3). Theorists have discussed the adaptive benefits gained by successfully responding to stressors (Ozbay et al., 2007; Rutter, 2012). Evidence from human and animal models suggests that controlled exposure to stressors expands the dynamic range of social homeostasis, fostering resilience and behavioral flexibility. Cold pressor tests, where participants briefly submerge their arm in ice water for 3 min, improves selective attention and episodic memory on the Eriksen flanker task, in which participants have to respond only to the central target while ignoring surrounding “flanker” stimuli (Shields et al., 2019; 2017). The phenomenon that brief, mild stressor exposure enhances subsequent coping capacity by stimulating, but not overwhelming neurophysiological mechanisms has been termed “stress inoculation” (Table 1; Meichenbaum, 1985; Meichenbaum and Novaco, 1985).

In humans, stress inoculation therapy is a form of CBT that consists of three phases: education about the stressor of interest, acquisition and rehearsal of coping skills, and gradual application of those skills to increasingly challenging stressors, similar to exposure therapy (Meichenbaum, 1985; Foa et al., 1999; Adenauer et al., 2011). Stress inoculation therapy has been shown to reduce rates of anxiety, depression, and PTSD symptoms while improving attention and reappraisal through enhanced top-down processing of aversive stimuli (Lustman and Sowa, 1983; Manderino and Yonkman, 1985; Foa et al., 1991; Saunders et al., 1996; Adenauer et al., 2011; Lueken et al., 2014; Kashani et al., 2015; Hourani et al., 2016; Stange et al., 2017; Jackson et al., 2019). Functional magnetic resonance imaging (fMRI) reveals these changes are mediated by corticolimbic circuits that re-evaluate threatening stimuli (i.e., photos of fearful and angry faces) before triggering the stress response indexed by blood cortisol levels (Flor, 2014; Comte et al., 2016; Smail et al., 2020; Kim and Kim, 2021). Developmentally, moderate early life stress fosters future stress adaptation (Hostinar and Gunnar, 2013; Ellis and Del Giudice, 2019; Szepsenwol, 2022). In addition, children in environments with moderate, unpredictable stress exhibit better emotional regulation, enhanced cognitive flexibility, and lower anxiety disorder risk (Holmes, 1935; Boyce and Chesterman, 1990; Crnic and Greenberg, 1990; Rutter, 2012).

In monkeys, stress inoculation typically involves brief, intermittent bouts of maternal separation (six 1 h episodes), which subsequently enhance voluntary object exploration, reduce distress vocalizations, and lower blood cortisol (Coe et al., 1983; Hennessy, 1986). Neuroimaging demonstrates that stress inoculation in these animals increases dopamine receptor availability in ventral striatum, putamen, and caudate nucleus (Parker et al., 2004; Lee et al., 2016), increases vmPFC volumes with white matter myelination (Katz et al., 2009), and leads to hippocampal neurogenesis (Lyons et al., 2010). These adaptations correspond to reduced basal corticotropin and attenuated HPA axis responsiveness (Lyons, 2009; Lyons et al., 2010). In rodents, repeated maternal separations attenuate corticosterone release to novel stimuli and improve coping behaviors in stress challenges such as restraint, exposure to novel environments, and mild footshock (Levine, 1967; Coe et al., 1983; Meaney, 2001). Other rodent stress inoculation models using modified social defeat paradigms where the subject undergoes 15 min of exposure to an aggressive resident mouse once a day for 7 d yield greater social interaction with an unfamiliar conspecific, more active defense during social defeat, and enhanced extinction of conditioned fear (Brockhurst et al., 2015; Ayash et al., 2020, 2023). Social play is a form of enrichment that is another example of mild, intermittent stress that expands dynamic range (Fig. 3). While enrichment is often conceptualized as the opposite of stress, many enriched social environments involve arousal, unpredictability, and challenge, features that engage mechanisms similar to those recruited by stress inoculation, and has led to the “stress inoculation theory of social enrichment” (Crofton et al., 2015). Play is observed across mammalian species and can function as “practice” for adult skills including complex social interactions (Allen and Bekoff, 1994; Pellegrini and Smith, 1998; Weisberg, 2015). In chimpanzees, the frequency and vigor (vigorous play has sequences involving chasing, wrestling, and mock fighting) predicts successful integration into future social groups, dominance, and reproductive success (Poirier and Smith, 1974; Graham and Burghardt, 2010; Berghänel et al., 2015). In rodents, rough-and-tumble play reverses stress-induced deficits in forced swim, novelty-induced hypophagia, and sucrose preference tests (Burgdorf et al., 2017), while play deprivation reduces prosocial behavior, increases aggression, and impairs cognitive flexibility (Panksepp and Beatty, 1980; Marquardt et al., 2023).

While brief, intermittent stress can broaden the dynamic range, both insufficient and excessive stress narrow the dynamic range of social homeostasis (Fig. 3). In our model, environments lacking in variety of social experiences, such as chronic social isolation (which is known to produce aggression, social avoidance, and antisocial behavior; Lee et al., 2021), result in narrowing of the dynamic range and a lowering of the set-point such that reintroduction to the individual’s previous optimum is subsequently perceived as a surplus given the change in set-point and/or dynamic range. In humans, early social deprivation delays executive function and language development (Nelson et al., 2007; McLaughlin et al., 2014). In adulthood, loneliness is linked to tonic alertness, accelerated cognitive decline, and abnormal connectivity in networks for social cognition and emotion regulation (Cacioppo et al., 2015; Layden et al., 2017; Vitale and Smith, 2022). Solitary confinement produces neural changes akin to physical pain, including hippocampal atrophy, EEG disruptions, and shrinkage of cortical neurons (Strong et al., 2020). Mice subjected to chronic social isolation demonstrate signs of hypervigilance, showing increased aggressiveness and hypersensitivity to threatening stimuli (Zelikowsky et al., 2018). Rhesus monkeys reared in prolonged social isolation develop stereotypies, hypervigilance, and exaggerated fear or avoidance upon reintroduction to conspecifics (Harlow and Suomi, 1971). Adults removed from stable groups show heightened startle responses, vigilant scanning, and elevated cortisol upon reuniting with a social group (Capitanio et al., 1998). Across species, these findings suggest that insufficient stress yields a hypersensitive system, where minor challenges elicit disproportionate distress, indicating narrowing of the dynamic range.

At the opposite extreme, high stress overwhelms the systems of homeostatic maintenance and narrows dynamic range. Developmental trauma, including childhood maltreatment, combat, or other extreme adversity, often leads to neuropsychiatric disorders characterized by social dysfunction, such as post-traumatic stress disorder (PTSD), social anxiety, and attachment disorders (Shonkoff et al., 2009; Ventriglio et al., 2015; Targum and Nemeroff, 2019; Juruena et al., 2020). Neuroimaging in trauma-exposed individuals reveals reduced cortical thickness, disrupted white matter integrity, and hyperactive amygdala responses to threat cues (Tottenham and Sheridan, 2010; Dannlowski et al., 2013). Severe and chronic stress produces hippocampal atrophy, prefrontal dysfunction, and amygdala hyperactivity (Sapolsky, 2003; McEwen, 2017), and other reviews have found that it impairs behavioral flexibility (Hurtubise and Howland, 2017). Clinically, trauma-exposed individuals exhibit heightened hypervigilance, marked by exaggerated startle responses, increased environmental scanning, sleep disturbances, and chronic hyperarousal, even under neutral or safe conditions, reflecting a persistently narrowed threshold for perceived threat (Ogden et al., 2006; Center for Substance Abuse Treatment (US), 2014; Ressler et al., 2022). These findings illustrate that, like low stress, high stress yields a hypersensitive system that responds disproportionately to minor environmental fluctuations (Table 1).

## Conclusions and Future Directions

In this review, we extend our framework of social homeostasis by adding two central elements that had yet to be accounted for. First, we highlight the role of previous experience as a force that continuously reshapes the brain. Second, we propose that this history calibrates the dynamic range, informing how individuals respond to dynamic social environments. Here, we provide a testable model for investigating how experience modifies the detector, control center, and effector nodes of social homeostasis throughout the lifespan.

Beyond the individual, larger social contexts play a critical role in calibrating the dynamic range. Across cultures, infant care and early social experience vary. For example, infants in hunter-gatherer societies spend up to 90% of their time in physical contact with an adult or older child, compared with 18% in a Western sample (Hewlett et al., 1998; Hewlett and Lamb, 2002). These infants nurse around four times per hour in short bouts, sleep in contact with their mothers, and are alloparented by nonparental caregivers for 40–50% of their childhoods (Konner, 2005; Chaudhary and Swanepoel, 2023). In contrast, Western caregiving often involves scheduled feeding approximately every 2–4 h, solitary sleep, and caregiving by one or two primary caregivers (Nelson et al., 2001; Willinger et al., 2003; Hauck et al., 2008; Iacovou and Sevilla, 2013). Because of these different practices, Western infants are more likely to experience longer, more frequent bouts of unsoothed crying than hunter-gatherers or others practicing similar care (Barr et al., 1991; Hewlett et al., 1998; St James-Roberts et al., 2006). These caregiving practices likely calibrate parameters of social homeostasis in distinct ways, each adaptive in its respective cultural niche. Regarding the set-point, infants in hunter-gather societies are in constant contact with multiple individuals and are likely to have higher homeostatic set-points than those in western societies. The question of dynamic range is more complex.

Hunter-gatherer infants have more varied social experiences (i.e., alloparenting, exposure to more caretakers) than Western infants. In this respect, hunter-gather infants would tend toward a broader dynamic range than Western infants, along with a higher set-point for basal social contact.

Western infants that experience longer, more frequent bouts of unsoothed crying may have increased effector system activation (e.g., crying) to rectify the perceived deficit in social contact. Based on our model, we would predict that prolonged, frequent increases in effector system activation (active coping) will result in one of two adaptations to reduce the energy emitted. One possible solution is to adjust the social homeostatic set-point to expect less social contact, and another solution is to widen the dynamic range of tolerated social contact. In a case where the social environment is highly predictable (e.g., sleep training), the set-point may be reduced as infants develop self-soothing skills. In a case where the social environment is more variable (e.g., a child goes to daycare with a group, comes home to single caretaker, and sleeps alone in a crib), the individual may develop a wider dynamic range of tolerance.

However, in the case of overstress, the individual adapts by *extreme* lowering of set-point and *narrowing* dynamic range, becoming less tolerant and/or over-reactive to variations in environment. Predictable variability can effectively expand dynamic range, but unpredictable or extreme variability can induce trauma and a greater energy expenditure to control social environments into a narrow range. If the tolerable range is exceeded in excess, then our model predicts that the individual becomes *less tolerant* to perturbations in social world and the dynamic range actually narrows, in a manner akin to stress sensitization (e.g., alcoholic mom that traumatized her child could result in traits associated with borderline personality disorder such as hypersensitivity (e.g., fear of abandonment) or hyperreactivity (e.g., rage or suicidality) in response to social cues; Fig. 3).

In all cases, the accumulated social experiences of an individual from infancy to adulthood should provide neuroadaptations that best equip the individual for a social environment that shares the sensory statistics (quantity, quality, and variability of social contact) of their cumulative history of experiences. Considering larger cultural influences highlights that dynamic range plasticity is shaped not only by individual experiences, but also by broader social contexts, underscoring the need to incorporate these conditions into future models of social homeostasis.

Future research should work to disentangle the neural mechanisms underlying set-point shifts from those underlying dynamic range plasticity in social homeostasis. While both mechanisms likely shape social homeostasis, it remains unclear whether they rely on overlapping or distinct neural mechanisms. Progress will require longitudinal designs that establish reliable behavioral baselines, track how individuals respond to controlled social challenges across time, and pair these trajectories with circuit-level measures. Combining developmental manipulations in animal models with advanced neuroimaging and physiological monitoring in humans will help reveal whether changes in baseline preference (set-point) can be distinguished from changes in tolerance to changes in the environment (dynamic range).

Critically, these efforts must incorporate prior experience as a core variable. Factors such as housing density, cage complexity, social rank, and prior isolation in animal studies, or caregiving environment, socioeconomic context, and cultural norms in humans, profoundly shape tolerance. Systematic reporting of these variables, combined with emerging tools such as continuous home-cage monitoring and multi-animal tracking platforms (e.g., SLEAP; Pereira et al., 2022), would enable construction of “social diaries” that capture the cumulative pattern of experiences shaping behavior. By accounting for previous history into both experimental design and clinical assessment, we can begin to test how dynamic range plasticity emerges, how it gates set-point shifts, and how calibrated environmental challenges might be harnessed to foster resilience across dynamic environments.
